# Richmond Academy of Medicine and Surgery

**Published:** 1902-01

**Authors:** 


### SOCIETY REPORTS.

### RICHMOND ACADEMY OF MEDICINE AND SURGERY.

Regular Meeting Held November 26, 1901, Stuart McGuire, President, in the Chair; Mark W. Peyser, M. D., Secretary and Reporter. Dr. Marvin E. Nuckols read a Paper on "Angina Pectoris." (See page 667.)

### DISCUSSION.

Dr. M. D. Hoge, Jr., said that the diagnosis of a typical case of angina pectoris was not difficult. As to the pathology in the majority of well marked cases, some obstruction of the coronary arteries was found; again an atheromatous degeneration of the base of the aorta could be frequently detected. In some few cases post-mortems presented no pathological changes to the naked eye. But the practical part of this disease was the treatment. The nitrites were the remedies used to the best advantage. In one case in his own practice, where amyl nitrite and other remedies had been employed without benefit, nitroglycerin at regular intervals for several months afforded marked relief. It was well to know that the continued use of nitroglycerin required constant increase in the size of doses. One one-hundredth of a grain was sufficient for but two or three weeks. Patients could stand astonishingly large amounts. Dr. Hoge reported a case of pseudo angina pectoris in a young girl due probably to hysteria.

Dr. J. S. Wellford said he had seen many cases of pseudo angina pectoris, but was satisfied he had not seen more than three or four cases of the true form. In one patient whom he had the opportunity of examining several times, he could never detect any sign of heart disease; but she died suddenly while at stool, probably from straining. The most diagnostic symptom was pain radiating down the left arm only as far as the elbow. He always regarded the disease as nervous and gave the nitrites, or chloroform or ether, the latter two to their stimulating stage only. If there were any accompanying heart disease he treated that also.

Dr. J. Allison Hodges said that he had come to the conclusion that all cases of angina pectoris were either neivous in origin, or due to lesions of the aorta or aortic valves, the last two being the most frequent and important causes. Text-books would lead one to believe that the attack was spasmodic and evanescent, but he had seen cases in which it would last indefinitely. He agrees with Dr. Hoge regarding the value of nitroglycerin. Amyl nitrite, chloroform or ether had never been of benefit in his hands. Morphine and atropine afforded the quickest relief, but were dangerous, in that their use led to the formation of the morphine habit. Nearly all of his cases, when seen, were already habitues. It was of greatest importance in treatment to immediately and absolutely change the method of life and regulate the diet of the sufferer. He did not take stock in the diagnosis of coronary disease before death.

Dr. William S. Gordon said that in diagnosing angina pectoris the first thing to do was to not confound it with aortic disease. It was difficult to explain death from it by post-mortem. He referred to a case seen by Dr. H. M. Taylor and himself, which was thought to be disease of the liver, the patient having glucosuria and albuminuria. He had pain in the epigastrium, but never in the arm. Answering a hurried summons, he found the patient dead. The man was intemperate and had arterio-sclerosis. In another case every time there was undue exertion pain occurred at the pit of the stomach. There was a pericardial friction murmur at the tip of the sternum, which accounted for some of the pain; but the patient, when lying down, had a mitral systolic bruit, and the pain then came in the right place. Recurring to his first statement, Dr. Gordon said that the remarks of previous speakers did not wholly apply to the subject under discussion. Was the cardiac pain due to spasm and pressure on the sensory nerves? In aortic trouble death was usually due to dilatation of the left ventricle. If neuritis were the cause of the pain, as argued by some, it would remain in the intervals between attacks; but in true angina the patients were then apparently well. It could not be due to dilatation and excess of pressure in cases in which the pulse was not affected. The sudden death must be due to dilatation and paralysis of the heart; mere pain did not kill.

The nitrites and iodides were the remedies to be used. Nitroglycerin acted by producing arterial dilatation, the vessels during the attack being spasmodically contracted. The agent did not dilate the heart. If it did the patient would die more quickly. He had seen several cases in which, owing to impaired nervous mechanism, the pulse became thready, and nitroglycerin administered, produced dilatation and saved life.

Dr. Hodges asked if Dr. Gordon did not, after all, come back to the point that the aorta was mainly at fault. Was there not an aortitis at the base of it all? Of course, the pains of aortic valve diseases and angina pectoris were entirely different.

Dr. Gordon in reply said that it depended upon the definition of the disease. If this included aortic trouble he agreed with Dr. Hodges; but he believed the disease ought to be considered to exist only when the post-mortem showed no anatomical lesions. He thought there was to a slight extent a similarity between it and epilepsy, but that they were not identical.

Dr. Nuckols, in closing the discussion, said he did not believe in the pseudo form of the disease; but he thought there was a difference in degree in true angina; and that there was always some organic lesion, though it might escape detection. Regarding dilatation, a person suffering and having mitral regurgitation was relieved when compensation failed.

### REPORTS OF CASES.

Dr. Hugh M. Taylor reported a case operated upon because of three interesting features it presented. The patient's face looked as though she had taken large amounts of nitrate of silver, the body, however, being naturally jaundiced. Her attending physician had given bichloride of mercury. The gall-bladder was enormously distended, reaching three inches below McBurney's point, and being perceptible from a distance. It was difficult to tell whether it was the gall-bladder or cystic enlargement of the kidney. Her physicians had tapped it three weeks before, and had drawn off a quantity of limpid serum, which was difficult to understand. The case was also interesting because in the absence of jaundice and hypertrophic sclerosis, he would have said it was an appendicular enlargement. Because of her age and appearance, he expected to find cancer of the liver; but there was none. A quart or more of muco-serous fluid with a little pus, and tinged with bile, was drawn off, and a large number of stones removed from the gall-bladder by a cholecystostomy. None were looked for in any of the tubes because of the age and precarious condition of the patient. He expected her to die from cholemia, but if she did not he would operate further.

